# Supplementary material for: COVID-19 testing systems and their effectiveness in small, semi-isolated groups for sports events
Source: PLoS One. 2022 Mar 29;17(3):e0266197. doi: 10.1371/journal.pone.0266197 (PMC8963550; doi:10.1371/journal.pone.0266197)
Supplement: Attachment — Submitted filename: COVID.pdf [file pone.0266197.s001.pdf]

COVID-19 testing systems and their effectiveness in small, semi-isolated groups for sports events

This paper investigates the dynamics of COVID-19 within two groups of professional players. There are several comments that needs to be addressed before it has been published.

1. Every players has been tested every two weeks, where is the parameter in the model representing this fact?
2. Line 133-135, when the detected individuals have been confirmed they have been removed from the group. Why are that? Where does it represent in the model? Does it affect the population numbers?
3. They are two groups. Is there any interaction between these two groups and do the authors analyse differently for transmission dynamics of each group?
4. The authors can discuss the following papers
   - An analysis of covid-19 transmission in indonesia and saudi arabia. Communication in Biomathematical Sciences. 2020
   - Modelling the transmission dynamics of COVID-19 under limited resources. Communication in Mathematical Biology and Neurosciences. 2020.
