# Supplementary material for: COVID-19 testing systems and their effectiveness in small, semi-isolated groups for sports events
Source: PLoS One. 2022 Mar 29;17(3):e0266197. doi: 10.1371/journal.pone.0266197 (PMC8963550; doi:10.1371/journal.pone.0266197)
Supplement: Attachment — Submitted filename: Responses.docx [file pone.0266197.s002.docx]

First of all, we would like to thank both reviewers for reading our paper. We are happy to find that both reviewers recommend our paper for publication after revision. We have revised our paper based on the comments by reviewers. Our response to the comments is as follows.

Due to the request by PLOS ONE, a source code and all data analyzed in the study were presented in an open repository. In the previous version, random numbers were generated by run1() from the Numerical Recipes in C (Press, Teukolsky, Vetterling and Flannery 1992, Cambridge University Press) for Monte-Carlo simulations. The code is a copyrighted material, and hence we changed the random generator to that of the standard C library in the source code in the repository. We’ve redone all the simulations with the new code, and found that the results were consistent with the previous ones (except Figure 1). However, because this is a stochastic simulation, all values are slightly different, and hence we updated all mean values in the text and figures in the revised version. Please find the comparison of figures at the end of this document.

Reviewer #1: COVID-19 testing systems and their effectiveness in small, semi-isolated groups for sports events

This paper investigates the dynamics of COVID-19 within two groups of professional players. There are several comments that needs to be addressed before it has been published.

We appreciate your constructive comments.

The comments in 1-3 seem to be questions arising from our lack of description. We solve the dynamics in a group (termed “team”) and we do not consider the dynamics in the general public. To make the point clear, we revised a sentence in page 6, line 124-128

OLD:

In reality, there is the chance that two individuals will be infected at the same time or that infections will be brought into the group twice at different times, but in this study we ignore such conditions for the sake of simplicity.

NEW:

In reality, there is the chance that two individuals will be infected at the same time or that infections will be brought into the group twice at different times*, and the chance depends on the number of infected individuals in the general public*, but in this study we ignore such conditions and *we do not consider the dynamics of the general public assuming that the prevalence in the general public is stable in the period we concern* for the sake of simplicity. (added sentence are shown in italic)

1. Every players has been tested every two weeks, where is the parameter in the model representing this fact?

There are no explicit parameters for testing. The effect of testing on the infectious disease dynamics depends on [1] test sensitivities and parameters for the sensitivities are shown in the text (line 202-204 ) and Table 2, and [2] reduction of infected individuals detected by tests. In the dynamics, the reduction is implemented by decreasing infected individuals minus detected individuals by testing.

2. Line 133-135, when the detected individuals have been confirmed they have been removed from the group. Why are that? Where does it represent in the model? Does it affect the population numbers?

I regret that the term "remove" were misleading. In the simulation, the detected infected individuals are quarantined while they are infected (the total number of individuals in the team are decreased while they are quarantined). We added a sentence to make the point clear as

ADDED SENTENCE:

quarantined and do not contribute to the further spread of the infection both in the team and in the general public until they get recovered (line 144-146).

All the term “removed” in the text were changed to “quarantined”.

3. They are two groups. Is there any interaction between these two groups and do the authors analyse differently for transmission dynamics of each group?

Since we consider the infectious dynamics in small group only, we do not consider an interaction among groups. We think that the revisions we've made so far solve the concern by the reviewer.

4. The authors can discuss the following papers

• An analysis of covid-19 transmission in indonesia and saudi arabia. Communication in Biomathematical Sciences. 2020

• Modelling the transmission dynamics of COVID-19 under limited resources. Communication in Mathematical Biology and Neurosciences. 2020.

Thank you very much for these information. We cited the second paper in Introduction (line 57-58) and the first paper in Discussion (line 466-468).

Reviewer #2: In this paper, the authors have quantitatively assessed the effectiveness of systems for COVID-19 testing in small groups of sport teams that are semi-isolated from the general population by countermeasures against infection. They have assumed two types of group, and the dynamics of infection within each group has modeled by using a compartment model of infectious disease. I think that the paper entitled "COVID-19 testing systems and their effectiveness in small, semi-isolated groups for sports events" is well-organized and the significance of the main ideas are attractive. However, it needs some revisions from the point of authors and readers to improve the quality of the paper. After these major revisions, I suggest that this paper can be accepted to publish in "PLOS ONE".

Thank you very much for your time to read our paper.

My other comments are as follows:

1. The authors have used “Figure” in Pages 10, 12 etc. to write some details. Since the word “Figure” are usually use to show plots, it is better to use other title for this end.

We agree that the point makes sense, but since “Figure” is specified to use for any graphics and paintings in the PLOS ONE guidance, and we would like to use the term.

2. Also, I consider the literature is not enough and that is why, I recommend the authors to refer to other recent works indexed in Web of Science, Scopus, Emerald, Cambrige, and of course MDPI Journals. For instance:

• Shokri, A., Mehdizadeh Khalsaraei, M., Molayi, M., Nonstandard Dynamically Consistent Numerical Methods for MSEIR Model, J. Appl. Comput. Mech., 8(1), (2022): 196-205.

• Mehdizadeh Khalsaraei, M., Shokri, A., Noeiaghdam, S., Molayi, M., Nonstandard Finite Difference Schemes for an SIR Epidemic Model, Mathematics, 9(23), (2021): 3082.

Thank you very much for these information. We have cited these papers (line 107-109) and other papers in the revised version.

With many thanks and best regards.

We too have many thanks and best regard to you.

Comparison of the old and new figures.

Figure 1

There was an mistake in the previous version. The total number of infected individuals were correct, but the number of presymptomactic at day 1 (P1) and at day 2 (P2), and symptomatic (Is) and asymptomatic (Ia) were wrong (Is were plotted as that of P1+P2, and number of P1+P2+Ia was plotted as that of Ia+Is). The mistake was corrected in the revised version.

OLD FIGURE1	NEW FIGURE1

Figure 2

OLD FIGURE 2

NEW FIGURE 2

Figure 3

OLD FIGURE 3	NEW FIGURE3

Figure 4

OLD FIGURE 4

NEW FIGURE 4

Figure 5

OLD FIGURE5

NEW FIGURE 5

Figure 6

OLD FIGURE 6

NEW FIGURE 6
